# Metal–Organic Framework as Contrast Agents for Magnetic Resonance Imaging

**DOI:** 10.3390/pharmaceutics18050621

**Published:** 2026-05-19

**Authors:** Weiqi Wang, Zijiao Yan, Yajie Yu, Mengjiao Zhou, Hejian Xiong, Tingting Liu

**Affiliations:** 1 School of Pharmacy, Nantong University, Nantong 226001, China; wwq1990@ntu.edu.cn (W.W.);; 2 Department of Medical Imaging, Affiliated Hospital of Nantong University, Nantong 226001, China; 3 Key Laboratory of Mental Health of the Ministry of Education, Guangdong-Hong Kong-Macao Greater Bay Area Center for Brain Science and Brain-Inspired Intelligence, Guangdong-Hong Kong Joint Laboratory for Psychiatric Disorders, Guangdong Province Key Laboratory of Psychiatric Disorders, Guangdong Basic Research Center of Excellence for Integrated Traditional and Western Medicine for Qingzhi Diseases, Department of Neurobiology, School of Basic Medical Sciences, Southern Medical University, Guangzhou 510515, China; hjxiong@smu.edu.cn

**Keywords:** metal-organic framework, magnetic resonance imaging, contrast agent, biomedical diagnosis, theranostic application

## Abstract

Metal–organic frameworks (MOFs) possess unique structural tunability, abundant coordination sites, and outstanding biosafety, rendering them highly advantageous for the development of high-performance magnetic resonance imaging (MRI) contrast agents. In light of the significant advancements in MOF-derived theranostic platforms, a comprehensive overview focusing on their classification and clinically oriented applications is urgently required. This review provides an in-depth examination of various categories of MOF-derived contrast agents, including T1, T2, dual-mode, ratiometric and 19F imaging systems, and analyzes the correlation between structural characteristics and imaging performance. Furthermore, it highlights typical MRI-guided therapeutic applications, such as those related to atherosclerosis, bacterial infections, and cancer immunotherapy. The review systematically addresses existing challenges, including issues related to biodegradability, metabolic behavior, and biosafety. It also summarizes the rational design principles for novel MOF contrast agents, aiming to facilitate their transition from fundamental research to clinical applications.

## 1. Introduction

Molecular imaging technologies have provided important guidance for comprehending disease mechanisms, identifying new pharmacological targets, and evaluating treatment responses [1,2,3]. In clinical practice, computed tomography [4,5,6], magnetic resonance imaging (MRI) [7,8], as well as positron emission tomography [9,10], have become indispensable diagnostic tools in clinical practice. In particular, MRI represents a non-invasive diagnostic tool characterized by excellent soft-tissue contrast, high spatial resolution, non-ionizing nature, and deep tissue penetration capability [11,12]. Since the late 1980s, the use of MRI for quantitative flow imaging has become a standard practice in cardiothoracic and vascular MRI, enabling the assessment of hemodynamic abnormalities in patients with cardiovascular disease [13,14,15]. Despite technological advancements, MRI still lacks sufficient sensitivity for detecting low-abundance biomarkers in pathophysiological lesions [16,17].

To improve imaging effectiveness, contrast agents are commonly utilized to augment the signal contrast between normal tissues and pathological lesions [18,19,20]. Early contrast agents like Gd-DTPA, approved by the US Food and Drug Administration (FDA) in 1988, exhibit extracellular distribution and short rotational correlation times, leading to low relaxivity (r1 = 4.87 mM^−1^s^−1^ at 1.5 T) [21,22]. Superparamagnetic iron oxide nanoparticles (SPIONs) gained increasing attention in the 2000s, providing T2-weighted contrast through superparamagnetic relaxation enhancement [23,24]. However, conventional MRI contrast agents, either gadolinium-based chelates or SPIONs, encounter significant limitations. Gadolinium-based chelates pose risks of nephrogenic systemic fibrosis in patients with impaired renal function and potential long-term brain retention [25,26], while SPIONs suffer from low T2-weighted relaxivity, rapid clearance by the reticuloendothelial system (RES), and oxidative stress induced by iron ion leakage [27,28]. MRI contrast agents have evolved toward high sensitivity and specificity, with intelligent response to pathophysiological stimulation to enhance signal-to-noise ratio and improve diagnostic accuracy [29,30,31].

Owing to their tunable size and morphology, easy surface modification, excellent physicochemical performance, and favorable biocompatibility, functional metal–organic framework (MOF) nanomaterials have been extensively applied in the biomedical field [32,33,34]. MOFs, as crystalline porous materials constructed from metal ions/clusters and organic linkers, possess structural advantages that make them ideal candidates for MRI applications [35,36]. Notably, the clearance pathways of MOFs are strongly dependent on their particle size, surface properties, and degradation behaviors [37]. Specifically, MOF nanoparticles with a size of 10–100 nm are mainly cleared by RES, while ultra-small MOF nanoparticles (<10 nm) can be efficiently cleared through renal filtration [38,39]. Surface modification with hydrophilic materials can further reduce RES recognition, prolong their blood circulation time, and optimize tumor accumulation [40]. Compared to clinically approved MRI contrast agents, MOF-based contrast agents generally achieve much higher relaxivity through atomic arrangement of paramagnetic metal ions. As an example, Gd-MOFs synthesized with 1,4-benzenedicarboxylate and 1,2,4-benzenetricarboxylate exhibit an r1 of 83.9 mM^−1^s^−1^, approximately 17 times higher than that of commercially available Gd-DTPA [41].

Rational structure design or surface modification endows MOFs with suitable biocompatibility, avoiding premature leakage and ensuring sensitivity towards pathological stimulation [42]. The median lethal dose (LD_50_) of Gd-based MOFs generally exceeded 1000 mg/kg, which is much higher than the free Gd^3+^ (100–200 mg/kg) [43,44,45]. Typically, endogenous ligands and nutritional metal ions are considered relatively safe, which renders them preferable for constructing MOF-based MRI contrast agents with suitable biocompatibility [46]. Additionally, MOFs possess inherent stimuli-responsive characteristics that facilitate lesion-specific signal enhancement and improve the signal-to-noise ratio [47]. The T1 relaxivity of Mn(III)-TCPP significantly increases upon glutathione (GSH) stimulation, with r1 values increasing from 2.65 mM^−1^s^−1^ to 6.02 mM^−1^s^−1^ [48]. To further optimize the performance of MOF-based contrast agents, several strategies can be adopted. First, paramagnetic metal ions can be incorporated into MOFs via ion exchange [49,50] which is particularly useful for inert MOFs with high structural stability, ensuring crystallinity and avoiding metal ion leakage. In addition, MOFs hybridized with multiple metal nodes or post-modification offer multimodal or ratiometric contrast, enabling tailoring of relaxivity for specific imaging applications. For MRI applications, MOFs with modular metal ion incorporation properties allow for dense concentration of paramagnetic metal ions to ensure suitable relaxivity. Surface modification strategies could be optimized to reduce metal ion leakage, clarify biodistribution patterns, and establish standardized long-term toxicity evaluation protocols [51]. Ongoing innovations in green synthesis and surface engineering will further enhance the biosafety, biocompatibility, and stability under physiological conditions [52]. Moreover, establishing standardized long-term toxicity evaluation protocols and clarifying the biodistribution and biodegradation is essential.

**Scheme 1:**
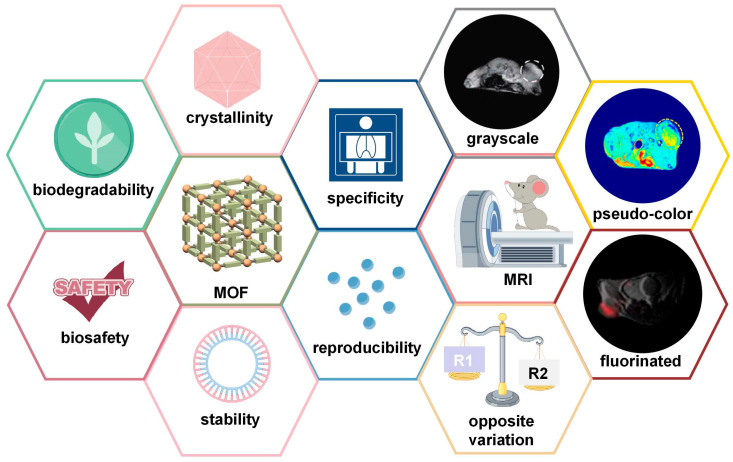
Schematic overview of MOF-based MRI contrast agents for theranostic applications. The diagram highlights core material properties (crystallinity, stability, biodegradability, and biosafety) and diverse imaging modalities (grayscale, pseudo-color, fluorinated, and dual-mode ratiometric contrast). Specificity and reproducibility are depicted as central links connecting MOF structural design to imaging performance, reflecting the structure–function correlation in these theranostic systems. Reproduced from [53]. Copyright 2026 Wiley-VCH GmbH; reproduced from [54]. Copyright 2024 ACS publication; reproduced from [55]. Copyright 2023 ACS publication.

Benefiting from their adjustable porosity, excellent metal ion loading performance, and customizable surface properties, MOFs are compelling candidates for theranostic applications combined with high-resolution MRI. Although remarkable advancements have been made in optimizing the structure and enhancing the performance of MOF-derived MRI contrast agents, most current studies focus primarily on material synthesis and preliminary imaging evaluation, without a comprehensive overview covering the rational classification, performance verification, and clinical translation process. In this review, we first clarify the classification of MOF-based contrast agents and highlight representative examples of T1, T2, dual-mode, ratiometric, and 19F MRI systems (Scheme 1). We then summarize state-of-the-art MRI-guided theranostic applications, including atherosclerosis management, antibacterial therapy, and epigenetic MOF-mediated metalloimmunotherapy. Key factors governing imaging performance and therapeutic efficacy are also discussed in depth. Finally, critical challenges toward clinical translation are identified and future perspectives are proposed, with the purpose of providing insightful guidance for practical biomedical applications of advanced MOF-based theranostic nanoplatforms.

## 2. Classification of MOF-Based Contrast Agents

Paramagnetic or superparamagnetic elements, such as Gd, Mn, and Fe, are the core components determining the imaging performance of MOF-based contrast agents. These metal ions exert their contrast effects by regulating proton relaxation rates, and the type of metal ion incorporated into the MOF directly dictates the MRI weighting mode. Specifically, Mn-MOFs and Gd-MOFs are commonly used for T1-weighted imaging due to their strong paramagnetism, while Fe-MOFs are ideal for T2-weighted imaging owing to their superparamagnetic properties. Detailed information regarding the composition, imaging type, key characteristics, and relaxivity of various MOF nanoformulations employed as MRI contrast agents is provided in Table 1.

### 2.1. T1 Contrast Agents

Traditional T1-weighted contrast agents mainly comprise paramagnetic metal chelates, which can significantly shorten the T1 relaxation time and achieve efficient contrast enhancement [65,66,67]. MOFs coordinated with paramagnetic metal ions emerged as promising candidates for MRI, owing to their adaptable structures, high relaxivity, and multifunctional capabilities. In comparison to clinically used gadoteridol, host–guest nanoplatformulation of β-cyclodextrin-based MOF demonstrated ~5-fold higher T1 relaxivity along with improved pharmacokinetic profiles [68]. Encapsulating Gd-DTPA within the porous framework of MOF-808 yields an r1 relaxivity of 30.1 mM^−1^s^−1^ (0.5 T), 5.4-fold higher than that of commercial Magnevis [69]. Post-synthetic functionalization endows the spn-type MOF high longitudinal relaxivity and allows for monitoring drug release with abundant accessible coordination sites [70]. Of note, the bottom-up assembly controlled by ultrasound produces Co-Mn-TCPP nanosheets with a high r1 relaxivity of 12.05 mM^−1^s^−1^, facilitating MRI-guided surgical removal of small tumor lesions [43].

**Figure 1:**
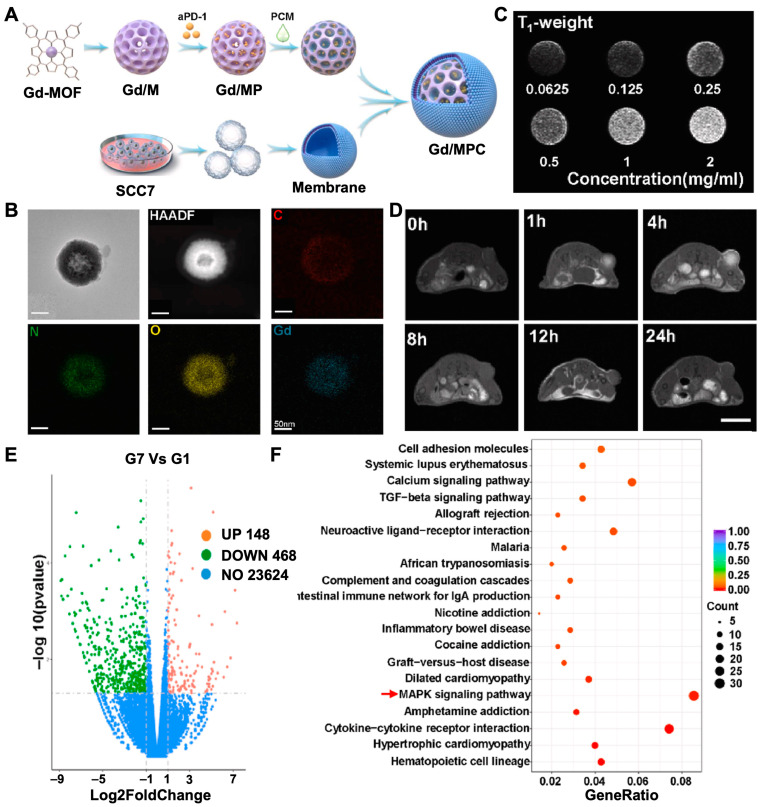
(**A**) Schematic overview of the fabrication strategy for Gd/MPC; (**B**) morphological features and elemental mapping characterization of Gd/MPC; (**C**) concentration-dependent T1-weighted MRI images in vitro; (**D**) T1-weighted MRI of tumor-bearing mice prior to and following Gd/MPC administration, scale bar: 1 cm; (**E**) volcano map and (**F**) KEGG analysis of Gd/MPC relative pathways (red arrows). Reproduced from ref. [72]. Copyright 2024. Available under a CC BY-NC-ND license.

Utilizing paramagnetic metal ions Gd^3+^ as inorganic construction units, Gd-MOFs hold great potential in increasing the accuracy of cancer treatments guided by T1-weighted MRI [71]. In this regard, cancer cell membrane camouflaged Gd-MOF (Gd/MPC) has been designed and fabricated for MRI-guided microwave (MW) hyperthermia therapy [72]. As depicted in Figure 1A, the pre-synthesized Gd-MOF was sequentially functionalized with anti-PD-1 antibody (αPD-1) and phase change materials (PCMs), then endowed with homologous targeting capability by coating with SCC7 cell membrane. Observed by transmission electron microscopy, the spherical Gd/MPC has an average particle size close to 182 nm, with C, N, O, and Gd uniformly distributed (Figure 1B). In the Gd/MPC nanoplatform, the loading content of αPD-1 was approximately 28.23%, as determined by UV–visible spectroscopy, and the content of 1-tetradecanol, a typical PCM, was calculated to be around 28% via thermogravimetric analysis. For Gd/MPC solutions, the enhancement of T1-weighted MRI signals was closely related to their concentration, showing a clear concentration-dependent pattern (Figure 1C). In C3H tumor-bearing mice, the maximum MRI signal intensity in tumor was found at 4 h following injection, which was in line with the intratumoral Gd^3+^ content quantified by inductively coupled plasma (Figure 1D). Under microwave exposure at 0.6 W/cm^2^ for 10 min, the intratumoral temperature rapidly rose to approximately 55 °C following Gd/MPC intravenous administration. Moreover, the synergistic integration of Gd/MPC with microwave hyperthermia and PD-1 blockage can effectively improve the antitumor effect by homologous targeting ability and significantly remodel the immunosuppressive tumor microenvironment. Figure 1E illustrates that 148 genes were up-regulated and 468 genes were down-regulated after Gd/MPC + MW treatment. Treatment with Gd/MPC + MW resulted in a marked downregulation of the MAPK-associated pathway, as evidenced by Kyoto Encyclopedia of Genes and Genomes (KEGGs) analysis, with MSK-2, c-Jun and c-Fos being the key affected molecules (Figure 1F). In addition, Gd/MPC undergoes gradual degradation in the acidic tumor microenvironment, enabling controlled drug release, thus holding great potential as a theranostic nanoplatform for cancer thermal ablation guided by MRI.

### 2.2. T2 Contrast Agents

**Figure 2:**
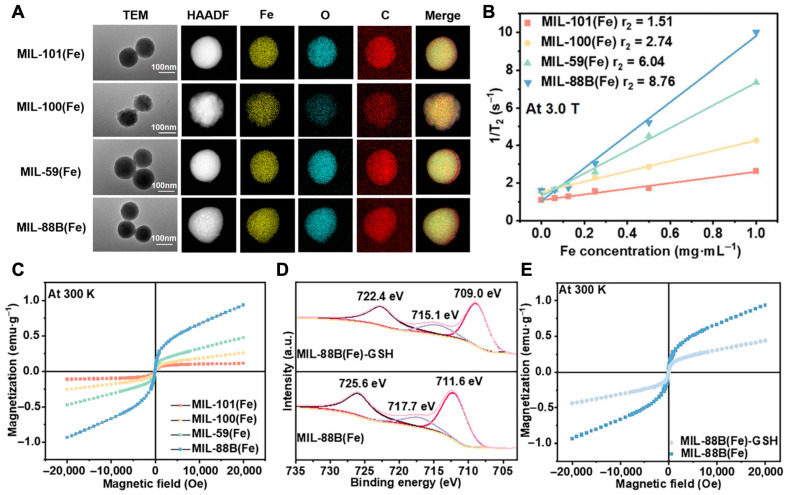
(**A**) Morphology and elemental mapping of MIL-88B(Fe), MIL-59(Fe), MIL-100(Fe), and (Fe)MIL-101(Fe); (**B**) T2 relaxivities monitored at 3.0 T magnetic field; (**C**) magnetic hysteresis measurements of four Fe-MOFs; (**D**) XPS and (**E**) magnetic hysteresis profiles of MIL-88B(Fe) in the absence and presence of GSH reduction. Reproduced from ref. [75]. Copyright 2025, the Royal Society of Chemistry.

T2 contrast agents provide negative contrast enhancement by shortening transverse relaxation times, which is distinct from T1 contrast agents, thereby leading to signal attenuation in MRI [73]. Owing to this negative contrast characteristic, differentiating such signals from the weak background of adjacent tissues can occasionally be challenging [74]. To gain a clearer insight into the magnetic resonance characteristics, four MOFs topologies were synthesized by using triangular Fe_3_O clusters as inorganic construction units [75]. As shown in Figure 2A, isotropic spherical nanostructures with an average size of approximately 150 nm were successfully constructed for MIL-88B(Fe), MIL-59(Fe), MIL-100(Fe), and MIL-101(Fe). Among these four Fe-MOFs with diverse topological structures, the Fe_3_O cluster densities were found to be 1.347, 1.115, 0.7098, and 0.3989 per 1000 Å3, and the T2 relaxivities were determined as 8.76, 6.04, 2.74, and 1.51 mL mg^−1^ s^−1^ at 3.0 T magnetic field (Figure 2B). By using the same metal-oxo clusters as magnetic lattices, MIL-88B(Fe) with an acs topological structure displayed the most prominent T2 relaxivity and saturation magnetization (Figure 2C). Moreover, this phenomenon can also be found under high magnetic field strengths or in Ga-doped MOF arrays. Notably, MIL-88B(Fe) exhibits a stable negative contrast effect when exposed to H_2_O_2_. Nevertheless, it shows reduced magnetic moments and fewer unpaired electrons under high GSH concentrations. X-ray photoelectron spectroscopy (XPS) verified that the satellite peak located at 715.1 eV corresponds to the generation of Fe(II) species, which arises from the reduction induced by excess GSH (Figure 2D). Meanwhile, magnetic hysteresis measurements verified that such a reduction reaction also leads to a gradual decline in saturation magnetization (Figure 2E). MIL-88B(Fe) is capable of discriminating M1 from M2 macrophages, thereby showing considerable potential for monitoring tumor development.

The unique structural features of Fe-based MOFs deepen the understanding of topology effects in materials of institute lavoisier (MIL) series framework, providing new insights into rational design of high-relaxivity T2 contrast agents. For proof of concept, solvothermally prepared MIL-53(Fe) achieves high T2 relaxivity (r2 = 50 mM^−1^s^−1^) and is capable of high dosage of drug loading and pH-sensitive drug release [76]. Moreover, hybridization of MIL-100(Fe) and ultra-small superparamagnetic iron oxide further enhances r2 relaxivity to 93 mM^−1^s^−1^ [77]. The variations in structure and performance across different Fe-based MOFs provide important insights for customizing T2 contrast agents applied in biological imaging and therapeutic interventions. Overall, this research deepens the understanding of MOF topological effects, which can be strategically utilized to optimize T2 relaxivity for biological imaging.

### 2.3. Dual-Mode Contrast Agents

Combining T1 and T2 MRI allows high-resolution tissue visualization and offers complementary diagnostic information [78,79]. Apart from employing paramagnetic metal ions as inorganic building units, organic linkers that integrate Fe^3+^ into porphyrin-based architectures can endow nanosystems with dual T1-/T2-weighted MRI capability. The optimization of MOF-based contrast agents mainly relies on structural adjustments; as an example, GT-MIL-88A can be engineered into monodisperse nanostructures with excellent MRI performance (r1 = 3.27 mg mL^−1^·s^−1^, r2 = 7.13 mg mL^−1^·s^−1^) [80]. Bimetallic Gd/FeMOF is endowed with dual-mode MRI capability, enabling imaging guided microwave thermal therapy and immuno-modulation [81].

**Figure 3:**
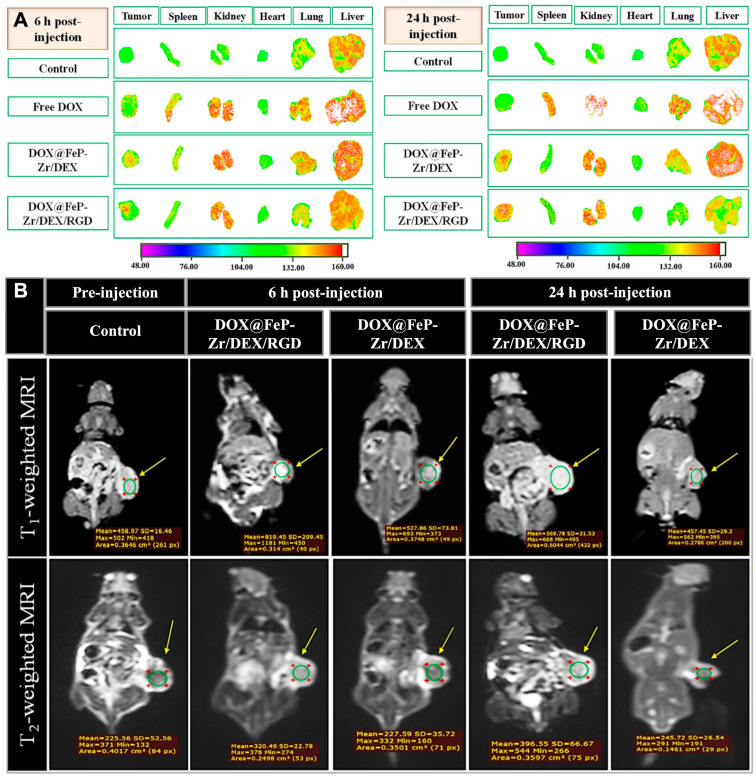
(**A**) Tissue distribution assessed on the basis of DOX fluorescence intensity at 6 h and 24 h post-injection; (**B**) coronal images of B16F10 tumor-bearing mice following the designated treatments. Reproduced from ref. [82]. Copyright 2025, the Royal Society of Chemistry.

As a paradigm, the Fe-porphyrin-based MOF denoted as FeP-Zr was synthesized via self-assembly employing Zr_6_ clusters as metallic nodes and tetrakis(4-carboxyphenyl)porphyrin as organic bridging ligands [82]. Then, DOX was encapsulated into FeP-Zr through electrostatic interactions, and the MOF was further surface-functionalized with RGD-dextran to enhance its biocompatibility and biostability, as well as active targeting ability. Compared to free DOX, the MOF-based drug delivery system significantly lowered the mean fluorescence intensity in major organs, whereas targeted modification with RGD further augmented the tumor-specific accumulation of the therapeutic agent (Figure 3A). Also, the content of DOX@FeP-Zr/DEX/RGD distributed in major organs at 24 h post-injection was considerably decreased compared with that in the 6 h group, suggesting gradual renal clearance. Consistent with biodistribution results quantified by fluorescence imaging, T1-/T2-weighted MRI at the tumor sites in B16F10-bearing mice reached a maximum at 24 h post-injection in both active targeting and non-target delivery systems (Figure 3B). In addition, incorporation of Fe^3+^ into the porphyrin structure has been proven to be beneficial for improving photothermal conversion and photothermal stability. Taken together, the DOX@FeP-Zr/DEX/RGD nanosystem demonstrates desirable targeted delivery, efficient photothermal conversion, and dual-mode MRI capability. The nanosystem with RGD-dextran surface modification achieved 90% tumor eradication when combined with 808 nm laser irradiation, with 4 out of 5 B16F10 tumor-bearing mice achieving complete tumor remission without recurrence, significantly outperforming non-targeted MOFs and free DOX.

### 2.4. Ratiometric Contrast Agents

Quantitative mapping of magnetic resonance relaxation times represents an advanced imaging approach that utilizes magnetic fields and radiofrequency pulses to enable high-precision visualization of in vivo pathophysiological alterations [83,84]. Ratiometric contrast agents improve detection accuracy by establishing a T1/T2 relaxation signal ratio, thereby normalizing signal intensity and eliminating interference from the pathogenic microenvironment. This strategy mitigates interference from variations in probe concentration, tissue perfusion, and instrument parameters. Additionally, their ratiometric readout enables real-time monitoring of target-specific biological processes and facilitates accurate differentiation between normal and pathological tissues by enhancing the contrast-to-noise ratio, which is crucial for improving the specificity and reliability of in vivo imaging diagnosis.

**Figure 4:**
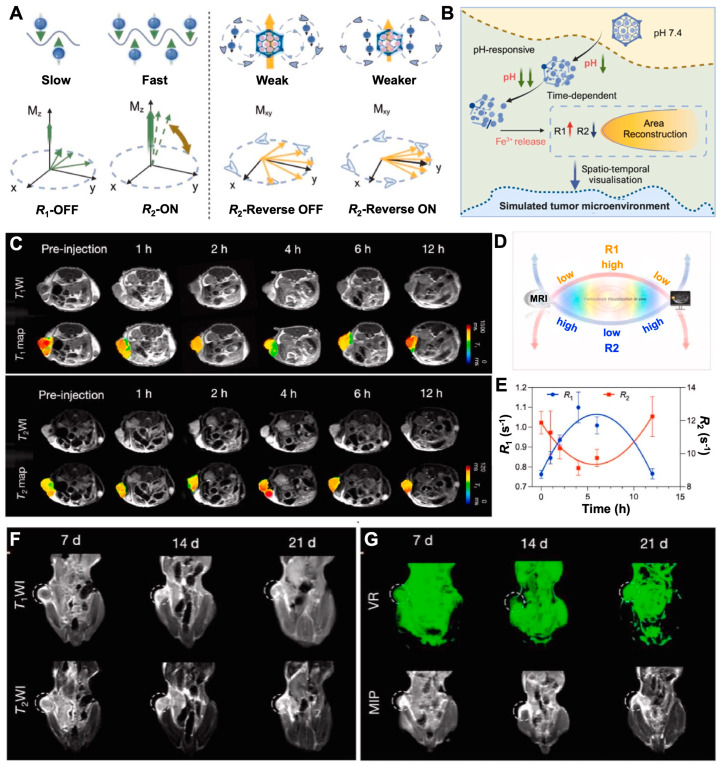
(**A**) Schematic illustration of ReMRT mechanisms in MOF-DOX nanoparticles; (**B**) pH-responsive MOF-DOX nanoparticles enabled spatiotemporal visualization of TME; (**C**) representative T1-weighted images and T2-weighted image in 4T1-bearing mice; (**D**) schematic illustration of Area Reconstruction; (**E**) quantitative analysis of increased R1 and decreased R2 relaxation; (**F**) coronal Gd-DOTA-enhanced images and (**G**) volume rendering and maximum intensity projection reconstructions after chemo-ferroptosis therapy. Reproduced from ref. [85]. Copyright 2024, Elsevier Ltd.

Recently, an Fe-MOF nanoplatform featuring reverse magnetic resonance tuning (ReMRT) performance was constructed to achieve real-time monitoring of ferroptosis-mediated tumor sensitization therapy [85]. Mechanistically, acidic conditions induce the disassembly of the ReMRT nanoprobe, which accelerates T1 relaxation by strengthening proton exchange and spin fluctuations, while simultaneously prolonging the T2 relaxation time (Figure 4A). This pH-responsive behavior synergistically enhances T1 and T2 imaging performance, and the “Area Reconstruction” algorithm further improves the accuracy of imaging evaluation (Figure 4B). According to T1-/T2-weighted images and mapping results, significantly enhanced signals were observed at 4 h post-injection, and then gradually declined due to probe degradation and metabolic clearance (Figure 4C). The use of “Area Reconstruction” method, the crossing points were approximately at 2 h and 10 h, with signal peak at 6 h identified as the optimal window for measuring ferroptosis-related biomarkers (Figure 4D,E). Consistent with MRI observations, the content of iron ions in the tumors was notably elevated at 4 h after injection. After chemo-ferroptosis therapy, Gd-DOTA-enhanced images revealed distinct tumor regression and necrosis at day 21 (Figure 4F,G). Notably, these pH-responsive T1/T2 contrast results highlight the potential of ReMRT nanoparticles to track real-time ferroptosis responses at the molecular scale, and achieve an approximately 95.1-fold higher sensitivity compared with conventional r1/r2 values.

### 2.5. 19F Contrast Agents

**Figure 5:**
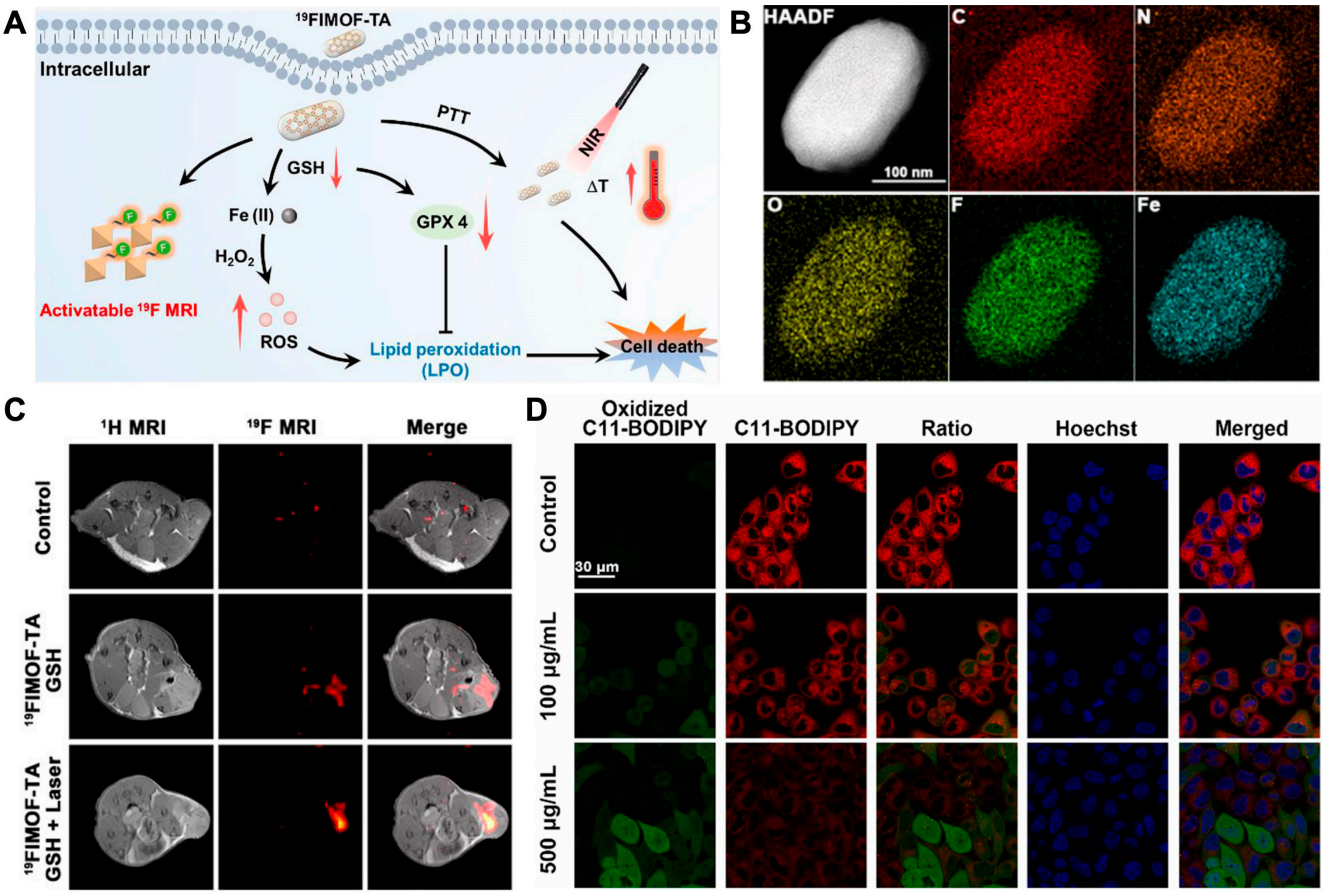
(**A**) Depiction of 19FIMOF-TA nanosystem with GSH responsiveness for switchable 19F MRI and combined ferroptosis-photothermal therapy; (**B**) high-angle annular dark-field STEM characterization and associated corresponding elemental mapping analysis of 19FIMOF-TA; (**C**) representative 1H and 19F MR images in HepG2-bearing mice; (**D**) LPO staining of HepG2 cells treated with 19FIMOF-TA. Reproduced from ref. [88]. Copyright 2023, American Chemical Society.

Fluorine-19 MRI is an evolving imaging modality with negligible background interference, which enables non-invasive and longitudinal insights into biological processes [86,87]. The MOF-based contrast agent permits long-term blood circulation and high-sensitive imaging [55]. Moreover, they possess high fluorine loading capacity and superior signal stability, ensuring imaging resolution during long-term monitoring. A tumor microenvironment responsive nanoprobe, 19FIMOF-TA, was rationally constructed using Fe^3+^ and fluorinated ligands, endowing it with activatable 19F imaging capability and enhanced tumor therapeutic efficacy (Figure 5A) [88]. As shown in Figure 5B, 19FIMOF-TA displays the spheroid shape with consistent dimensions of around 200 nm in length and 150 nm in width. In response to intratumoral GSH, these 19FIMOF-TA nanoparticles release fluorinated ligands and ferrous ions, thereby boosting the Fenton reaction, enhancing 19F MRI signals, and intensifying photothermal conversion efficiency (Figure 5C). Additionally, the ferrous ion supplement also facilitated ferroptosis execution, accompanied by increased cellular reactive oxygen species (ROS), exaggerated lipid peroxidation (LPO), and downregulated glutathione peroxidase 4 (GPX4) (Figure 5D). These findings reveal the great feasibility of the MOF-based 19F nanotheranostic agent integrating imaging diagnosis and therapeutic functions to realize synchronous monitoring and treatment of tumors for improved therapeutic outcomes. Furthermore, the tumor microenvironment-responsive activation feature ensures high specificity for tumor tissues, which reduces off-target effects and enhances safety of cancer therapy.

## 3. MOF-Based MRI Diagnosis and Atherosclerosis Treatment

**Figure 6:**
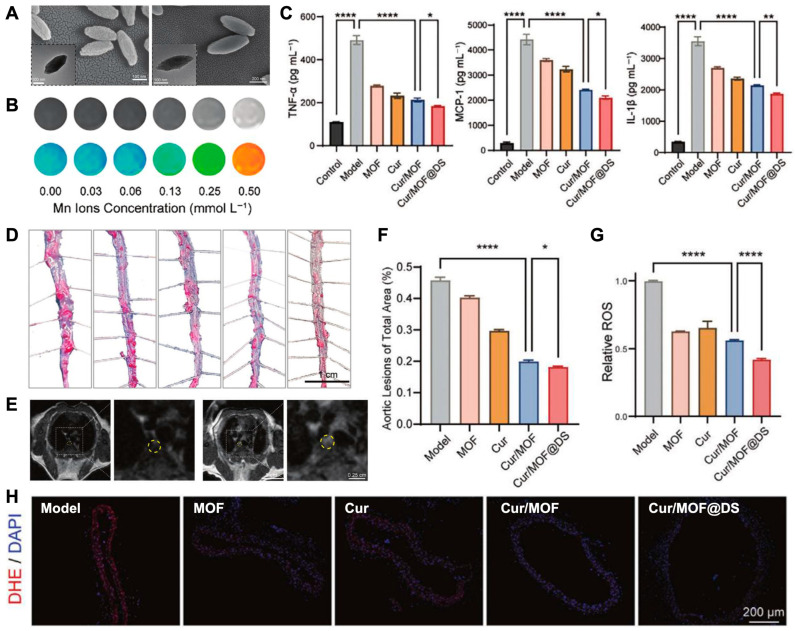
(**A**) Representative SEM and TEM (inset) images of MOF and Cur/MOF@DS; (**B**) MRI with varying Mn concentrations (3.0 T); (**C**) levels of the inflammatory cytokines MCP-1, IL-1β, and TNF-α levels in Raw264.7 cells under different treatment conditions; (**D**) oil red staining of aortic bulk after Cur/MOF@DS treatment; (**E**) representative T1-weighted images of aorta; quantitative analysis of (**F**) aortic lesions and (**G**) ROS accumulation within aorta; (**H**) DHE staining of aortic tissue after indicated treatment. Data were presented as means ± SD (n = 3). * *p* < 0.05, ** *p* < 0.01, **** *p* < 0.0001. Reproduced from ref. [96]. Copyright 2023, American Chemical Society.

Atherosclerosis, a representative cardiovascular disorder, is mainly driven by oxidized low-density lipoprotein (ox-LDL) accumulation and sustained chronic inflammation [89,90]. Generally, the generation of ox-LDL within the arterial intima is recognized as the primary initiating factor, and subsequent phagocytosis of ox-LDL by macrophages through scavenger receptors further drives the pathological progression of atherosclerosis [91,92]. Curcumin demonstrates potential for atherosclerosis treatment by scavenging free radicals, inhibiting inflammation, and lowering blood lipid levels [93,94,95]. For atherosclerosis treatment, a nanozyme-like MOF equipped with curcumin was developed, enabling enzyme-mimetic catalysis for ROS scavenging and MRI diagnosis of plaque formation [96]. As shown in Figure 6A, PCN-222(Mn) takes on ellipsoidal shape, with dimensions of approximately 240 nm in length and 90 nm in width. Then, curcumin was loaded via pore adsorption and further surface-coated with dextran sulfate (DS) for targeted delivery via overexpressed scavenger receptor. Observed by scanning electron microscopy (SEM) and transmission electron microscopy (TEM), there is no significant change in the morphology and size distribution after post-modification.

As expected, the T1-weighted MRI signal exhibits a positive correlation with the content of paramagnetic manganese ions within the MOF structure (Figure 6B). The longitudinal relaxation rate (r1) of 33.71 mM^−1^s^−1^ was calculated, highlighting the significant potential of this material for early detection of atherosclerotic lesions. As shown in Figure 6C, the Cur/MOF@DS treatment markedly decreased the concentrations of atherosclerosis-related proinflammatory cytokines, such as monocyte chemoattractant protein-1 (MCP-1), interleukin-1 beta (IL-1β), and tumor necrosis factor-alpha (TNF-α). Mechanistically, Cur/MOF@DS could affect cholesterol metabolism by activating the LXRα/ABCA1 signaling pathway to increase the expression of cholesterol transporters. Histological staining demonstrated that, in high-fat diet-induced apolipoprotein E-deficient mice, Cur/MOF@DS remarkably decreased atherosclerotic lesion area and lipid content in aortic specimens (Figure 6D,F). Tail-vein injection of the Cur/MOF@DS nanoplatform led to a prominently elevated MRI signal in the aortic region, confirming its efficacy in vascular imaging and atherosclerotic plaque evaluation (Figure 6E). According to dihydroethidium (DHE) staining, the fluorescence signal in aortic tissue was the lowest in the Cur-/MOF@DS-treated group, indicating efficient ROS clearance and favorable anti-atherosclerotic effects mediated by this nanosystem (Figure 6G,H). Of note, Cur/MOF@DS not only exhibits robust therapeutic efficacy against atherosclerotic lesions but also holds great promise for early diagnosis via MRI enhancement in the plaque microenvironment. The Cur/MOF@DS nanoplatform exhibited superior anti-atherosclerotic effects in apolipoprotein E-deficient mice, reducing atherosclerotic lesion area and ROS levels in aortic tissue to a greater extent than non-modified Cur/MOF, while maintaining excellent biosafety.

## 4. MRI-Guided Antibacterial Treatment

**Figure 7:**
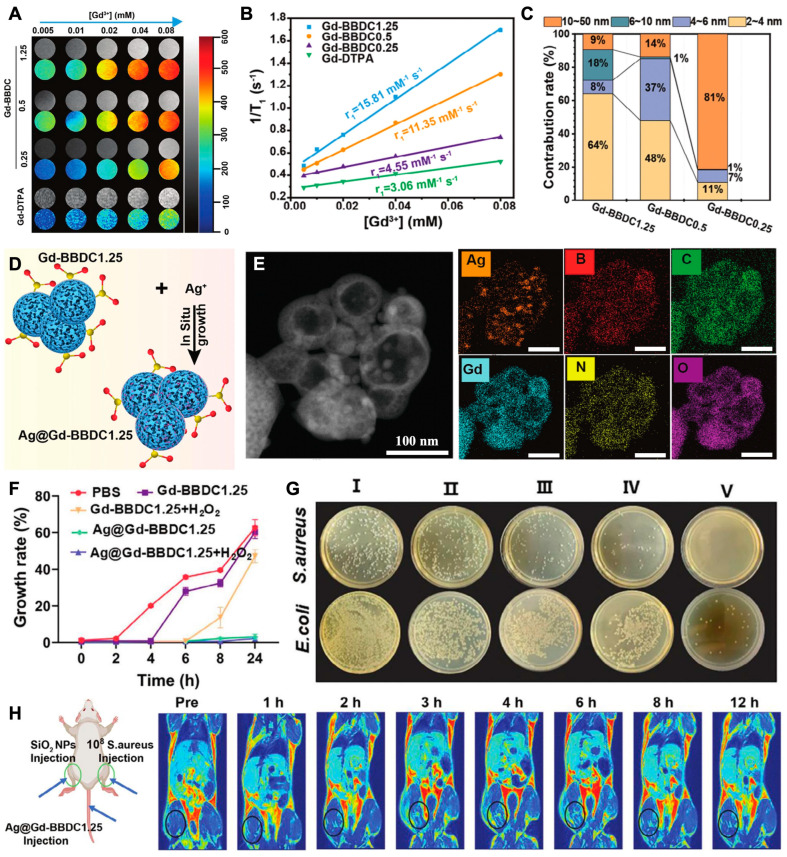
(**A**) T1-weighted MRI and (**B**) longitudinal relaxivity profiles of Gd-BBDC versus Gd-DTPA; (**C**) the distribution of pore sizes in Gd-MOF with different Gd^3+^/BBDC ratio; (**D**) schematic illustration of in situ reduction method to form Ag@Gd-BBDC1.25; (**E**) TEM image and corresponding elemental mapping of Ag@Gd-BBDC1.25 (scale bar = 100 nm); (**F**) the growth rate of *S. aureus* after indicated treatment; (**G**) typical photographic images of *S. aureus* and *E. coli* colonies, (I) PBS, (II) Gd-BBDC1.25, (III) Gd-BBDC1.25 + H_2_O_2_, (IV) Ag@Gd-BBDC1.25, (V) Ag@Gd-BBDC1.25 + H_2_O_2_; (**H**) representative T1-weighted images in *S. aureus*-induced myositis mice. Reproduced from ref. [102]. Copyright 2024, WILEY-VCH Verlag GmbH and Co. KGaA, Weinheim.

Antibiotic resistance emerging in bacterial pathogens toward traditional antimicrobial agents creates a critical challenge for the management of infectious disorders [97,98,99]. Nanomaterial-based treatments exhibit considerable potential in treating multidrug-resistant bacterial infections, as they can break through biofilm barriers and bypass conventional resistance mechanisms [100,101]. As a paradigm, Gd-MOF functionalized with 1-borono-3,5-benzenedicarboxylic acid (BBDC) as a bacteria-targeting moiety enables simultaneous bacteria-specific MRI diagnosis and in situ antibacterial treatment [102]. The Gd-MOF was synthesized via a one-step solvothermal approach, and the size uniformity and pore distribution were extensively optimized by tuning the Gd^3+^/BBDC ratio. As shown in Figure 7A, Gd-BBDC1.25 showed significantly stronger T1-weighted MRI contrast when compared to other Gd-MOFs with lower BBDC concentrations. Moreover, the longitudinal r1 monitored at a 1.41 T magnetic field is 5.16-fold that of commercially available Gd-DTPA (Figure 7B). Nitrogen adsorption and molecular dynamics simulations revealed that Gd-MOFs with different ratios of BBDC ligands resulted in differences in pore size distribution (Figure 7C). In comparison, the Gd-BBDC1.25 featuring an increased proportion of 2–4 nm mesopores more strongly restricts water molecule mobility, leading to enhanced longitudinal relaxivity. In situ reduction enabled the successful loading of silver nanoparticles (Ag NPs) into Gd-BBDC1.25, with its uniform particle size (~90 nm) and hierarchical porous structure well-maintained (Figure 7D,E). Impressively, the peroxidase (POD)-like activity of Ag@Gd-BBDC1.25 was notably enhanced, partly attributed to electron transfer across the formed Schottky heterojunctions. In addition, the presence of boronic acid groups from BBDC provides the nanoplatform with bacteria-targeting ability, which promotes the generation of toxic •OH via superior POD-like activity. In the presence of H_2_O_2_, Ag@Gd-BBDC1.25 induced a significant reduction in the OD600 value of bacterial suspensions (Figure 7F). The combined administration Ag@Gd-BBDC1.25+ H_2_O_2_ inhibited colony formation in both *Staphylococcus aureus* (*S. aureus*) and *Escherichia coli* (*E. coli*) (Figure 7G). In a myositis model, the signal intensity reached its maximum at 4 h following Ag@Gd-BBDC1.25 injection, indicating the targeted accumulation at infection sites. Of note, the T1-weighted MRI signal exhibited a quantitative correlation with *S. aureus*, showing a detection limit of approximately 10^5^ cfu. The Ag@Gd-BBDC1.25 demonstrates bacteria-targeting capability and improved catalytic performance, offering a general nanoformulation to overcome antibiotic-resistant bacterial infections via a bacteria-targeted, MRI-guided bactericidal modality.

## 5. MOF for MRI-Guided Cancer Treatment

MRI has become the cornerstone of modern diagnostic medicine, enabling non-invasive visualization of soft tissues with unparalleled spatial resolution [103]. By providing detailed anatomical and functional information of biological tissues, MRI has revolutionized non-invasive medical diagnostics, with applications in fields such as oncology, neurology, and cardiology [104,105]. MOFs have demonstrated exceptional utility in tumor imaging, leveraging their high relaxivity and capability for targeted delivery (Table 2). Surface modification of MOFs with PEGylation or cell membrane camouflage contributes to increased blood circulation duration and enhanced colloidal stability. In response to tumor acidity, MOF constructed with coordination bonds are prone to dissociation, thereby enhancing relaxivity by releasing paramagnetic ions. However, rapid degradation may induce systemic cytotoxicity or reduce imaging durability, emphasizing the critical requirements for precise structure design.

**Figure 8:**
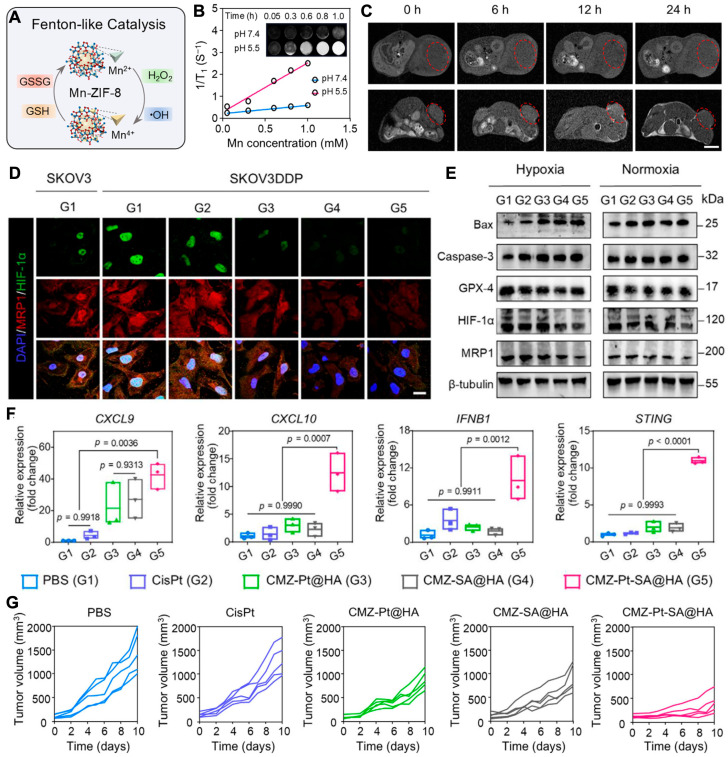
(**A**) Schematic illustration of Mn-ZIF-8 for GSH depletion and •OH generation; (**B**) the T1-weighted MRI and corresponding longitudinal relaxation rate (1/T1) for CMZ-Pt-SA@HA; (**C**) representative T1-weighted MRI images in mice; (**D**) representative CLSM images showing HIF-1α and MRP1 immunofluorescence in SKOV3DDP and SKOV3 cells (scale bar, 20 μm); (**E**) protein expression levels of MRP1, HIF-1α, GPX-4, Caspase-3, and Bax under normoxia and hypoxia; (**F**) relative gene expression of *IFNB1*, *CXCL9*, *CXCL10*, and *STING* following indicated treatments; (**G**) tumor volume growth curves in the patient-derived xenograft tumor model. Reproduced from ref. [125] Copyright 2024, WILEY-VCH Verlag GmbH and Co. KGaA, Weinheim.

In ovarian cancer, platinum serves as the standard chemotherapy in clinical settings, while acquired resistance persists as a significant therapeutic obstacle, promoting tumor recurrence and negatively impacting patient survival [119,120]. Platinum resistance arises from a diverse array of mechanisms, such as genomic and epigenetic modifications, DNA damage repair pathways, pharmacokinetic variations, metabolic reprogramming, and the tumor microenvironment [121,122]. Epigenetic modulator-mediated TME reprogramming can improve the sensitivity of platinum-resistant ovarian cancer to therapeutic interventions [123,124]. In this regard, MOF nanocomposite (CMZ-Pt-SA@HA) was designed and fabricated, enabling MRI-guided diagnosis and epigenetic regulation of cisplatin-resistant ovarian cancer [125]. As depicted in Figure 8A, the one-pot synthesized bimetallic MOF Mn-ZIF-8 integrates multiple functions, including the oxidation of GSH to oxidized glutathione (GSSG) and the decomposition of H_2_O_2_ into cytotoxic •OH. Owing to its pH responsiveness, CMZ-Pt-SA@HA exhibits markedly amplified T1-weighted MRI signals in acidic environments, showing a 4.2-fold enhancement relative to neutral conditions at a Mn^2+^ concentration of 1 mM (Figure 8B). T1-weighted MRI also revealed intensified signal enhancement in mouse models bearing patient-derived xenografts (Figure 8C). In SKOV3DDP cells, administration of CMZ-Pt-SA@HA resulted in a remarkable reduction in the fluorescence signals of HIF-1α and MRP1 (Figure 8D). Quantitative analysis revealed that fluorescence intensities were decreased by 43.1-fold and 66.5-fold in the CMZ-Pt-SA@HA group compared with the PBS control. Under normoxia as well as hypoxia, CMZ-Pt-SA@HA effectively inhibited the expression of GPX-4, HIF-1α, and MRP1, and promoted the expression of Caspase-3 and Bax (Figure 8E). Mechanistically, CMZ-Pt-SA@HA treatment enhances DNA damage, leading to activation of the stimulator of interferon genes (*STING*) pathway, accompanied by increased expression levels of *IFNB1*, *CXCL9*, *CXCL10*, and *STING* (Figure 8F). CMZ-Pt-SA@HA treatment effectively inhibits drug-resistant tumor progression in a highly aggressive patient-derived xenograft mouse model, with 70% of treated mice surviving beyond 30 days (Figure 8G). This MOF-based theranostic system overcomes drug resistance via the synergistic integration of epigenetic modulation, metalloimmunotherapy, and dual-modal NIR FL/MRI, highlighting its potent therapeutic efficacy against platinum-resistant ovarian cancer.

## 6. Conclusions and Outlook

MOFs represent a transformative and versatile platform for MRI contrast agents, offering unique advantages including modular structural design, superior proton relaxivity, inherent biocompatibility, and integrated theranostic capabilities. These distinctive features enable MOFs to outperform conventional MRI contrast agents, facilitating high-resolution imaging of pathological lesions and synchronous therapeutic interventions in diverse disease models, such as atherosclerosis, antibiotic-resistant bacterial infections, and platinum-resistant ovarian cancer. As summarized in this review, MOF-based contrast agents cover a full spectrum of MRI modalities, including T1, T2, dual-mode, ratiometric, and 19F imaging. Their utilization in MRI-guided theranostics presents exceptional advantages in elevating diagnostic precision and therapeutic outcomes, thereby establishing a robust basis for the development of precision biomedicine.

Despite substantial progress in MOF-based MRI contrast agents, their clinical translation continues to encounter formidable challenges. Key issues involve scalable synthesis with consistent particle size and performance, reproducibility between batches, and rigorous quality control. In addition, compliance with Good Manufacturing Practice (GMP), alongside the cost-effectiveness of raw materials and automated fabrication processes is still challenging. Optimizing continuous flow synthesis technology and intelligent process control may offer opportunities for scalable production of MOFs with uniform particle size, suitable stability, and batch-to-batch reproducibility. The FDA identifies MOFs as class II/III medical devices or drug-device combinations, necessitating extensive physicochemical characterization, biosafety evaluation, and clinical efficacy. The European Medicines Agency (EMA) enforces MDR 2017/745 compliance, emphasizing risk assessment, complete manufacturing documentation, and nanomaterial hazard mitigation. In brief, compliance with pharmacopeia standards calls for stringent oversight of heavy metal residues and endotoxin content, which makes MOF nanoparticulate formulations far from practical application despite remarkably improved imaging sensitivity. These challenges will eventually be addressed through ongoing technological advancements and interdisciplinary cooperation among nanotechnology, materials science, artificial intelligence, and clinical medicine.

## Figures and Tables

**Table 1:** Representative MOF-based nanoformulations as MRI contrast agents.

Metal	Ligand	Mode	Relaxation (mM^−1^s^−1^)	Morphology	Ref.
Mn(III)	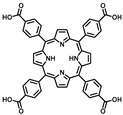	T1	r1 = 2.65 (without GSH); r1 = 6.08 (2.5 mM GSH)	fusiform-like structure: 170 nm in length, 50 nm in width	[48]
Mn(III)	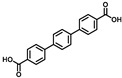	T1	r1 = 1.037 (without GSH); r1 = 7.650 (GSH treated)	rod-like structure	[56]
Mn(II)	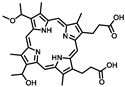	T1	r1 = 3.55 (PEGylated)	amorphous structure; spherical: 124 ± 51 nm	[57]
Mn(II)/Fe(III)	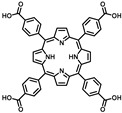	T1	r1 = 2.007	spindle-like structure: 100 nm	[58]
Fe(III)	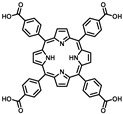	T2	r2 = 8.23 (PCN-600); r2 = 32.84 (PCN@PDA)	spindle: 150 nm	[59]
Fe(II)/Fe(III)	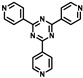	T2	r2 = 5.03 (pH 7.4); r2 = 9.27 (pH 6.0); r2 = 21.37 (pH 6.0 + H_2_O_2_)	hydrangea-like structure: 70 ± 11.5 nm	[60]
Mn(II)	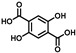	T1-T2	r1 = 7.3 in water; r1 = 66.4 in HSA; r2 = 55.3 in water; r2 = 121.2 in HSA	nanoparticle ranging from 50 to 150 nm	[61]
Gd(III)/Fe(III)	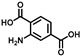	T1-T2	r1 = 6.03 in agar; r1 = 3.85 in water; r2 = 53.65 in agar; r2 = 26.72 in water	bipyramidal: 1.108 μm in length, 583 nm in width; spheroid: 229 nm	[62]
Zr(IV)/Mn(III)	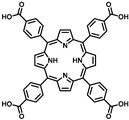	T1-T2	r1 = 30.3; r2 = 36.7 (0.5 T); r1 = 35.3; r2 = 42.5 (1.0 T)	spindle-like structure: 200 nm	[63]
Mn(II)	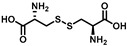	T1-T2	r1 = 4.98; r2 = 75.7	rod-like structure: 5 × 40 nm	[64]

Abbreviations: GSH, glutathione; HSA, human serum albumin; PCN, porous coordination network; PDA, polydopamine; PEG, polyethylene glycol.

**Table 2:** Brief summary of MOFs in the MRI-guided theranostic applications.

Materials	Metal	Ligand	Characteristic	Function	Models	Ref.
CPT/αPD-L1@Tpy-SS-MOF	Cu(II)	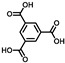	Antibody-gated; GSH response	T1-MRI/FL imaging/chemotherapy/checkpoint blockade	HepG2/AGS	[106]
CA@MOF-808(Zr/Fe)-AuNP-Mn-PEG	Zr(IV)/Fe(III)/Mn(II)	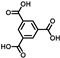	PEGylated; phosphate response	T1-MRI/CDT/chemotherapy	HepG2	[107]
NMOF-SNO	Zr(IV)/Mn(II)	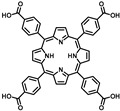	Heat/light response	T1-MRI/PTT/NO therapy	MCF-7	[108]
RGD-Au@Fe-MOF	Fe(III)	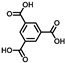	Tumor-targeted	T2-MRI/chemotherapy /PTT	MDA-MB-231	[109]
DOX@PCN@MnO_2_@PAH	Fe(III)	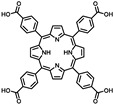	GSH response	T1-MRI/PDT/chemotherapy	4T1	[110]
Co/La-PB@MOF-199/GOx	Co(II)/La(III)/Cu(II)	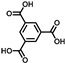	pH response/POD/CAT/GPx	T2-MRI/PTT/catalytic therapy	4T1	[111]
Fe-DOX@Gd-MOF-ICG	Fe(III)/Gd(III)	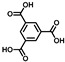	High drug loading ~71.4%; pH response	T2-MRI/PAI/chemotherapy/PTT/PDT	4T1	[112]
Mn^2+^&DOX@MOF	Mn(II)/Zr(IV)	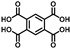	PEGylated; pH response	T1-MRI/chemotherapy	4T1	[113]
CH4T@MOF-PEG-AE	Fe(II)	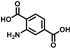	Tumor-targeted/BBB across	T2-MRI/FL imaging/PTT	U87	[114]
ICG@Mn/Cu/Zn-MOF@MnO_2_	Mn(II)/Cu(II)/Zn(II)	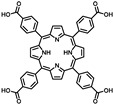	pH/light response/porous	T1-MRI/FL/PTT/PDT/CDT	U87	[115]
Mn-ZIF-8/5-Fu	Mn(II)/Zn(II)	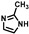	pH response	T1-MRI/chemotherapy	U87	[116]
FA-Hf-Mn-NMOF	Hf(IV)/Mn(III)	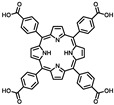	Tumor-targeted/high-Z elements/CAT	T1-MRI/CT/PAI/PTT/RT	S180	[117]
Mn_3_[Co(CN)_6_]_2_@MIL-100(Fe)@AS	Mn(II)/Co(III)/Fe(III)	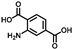	pH response/single/two-photon	T1/T2 MRI/chemotherapy/ROS therapy	HeLa	[118]

Abbreviations: 4T1, 4T1 murine breast cancer cell line; AGS, human gastric cancer cell line; AS, artesunate; BBB, blood–brain barrier; CA, carboplatin; CAT, catalase; CDT, chemodynamic therapy; CPT, camptothecin; CT, computed tomography; FA, folic acid; FL, fluorescence; GPx, glutathione oxidase; HeLa, Henrietta lacks cell line; HepG2, human hepatocellular carcinoma cell line; ICG, indocyanine green; MCF-7, Michigan cancer foundation-7; MDA-MB-231, MD Anderson-metastatic breast-231; NIR-II, near-infrared II; NO, nitric oxide; PAH, poly(allylamine hydrochloride); PAI, photoacoustic imaging; PDT, photo-dynamic therapy; POD, peroxidase; PTT, photothermal therapy; RGD, arginine-glycine-aspartic acid; RT, radiation therapy; S180, Sarcoma 180 cell line; SNO, S-Nitrosothiol; Tpy, terpyridine; U87, U-87 glioma cell line; US, ultrasound.

## Data Availability

No new data were created or analyzed in this study.

## Abbreviations

The following abbreviations are used in this manuscript:

MOFs	Metal–organic frameworks
MRI	Magnetic resonance imaging
SPIONs	Superparamagnetic iron oxide nanoparticles
DHTP	2,5-dihydroxyterephthalic acid
HSA	Human serum albumin
BBB	Blood–brain barrier
CDT	Chemodynamic therapy
CT	Computed tomography
FL	Fluorescence
NIR-II	Near-infrared II
NO	Nitric oxide
PAI	Photoacoustic imaging
PDT	Photo-dynamic therapy
PTT	Photothermal therapy
RT	Radiation therapy
US	Ultrasound
SEM	Scanning electron microscopy
TEM	Transmission electron microscopy
Gd/MPC	Cancer cell membrane camouflaged Gd-MOF
αPD-1	Anti-PD-1 antibody
PCM	Phase change materials
KEGGs	Kyoto Encyclopedia of Genes and Genomes
GO	Gene ontology
GSH	Glutathione
ReMRT	Reverse magnetic resonance tuning
ROS	Reactive oxygen species
LPO	Lipid peroxidation
GPX4	Glutathione peroxidase 4
ox-LDL	Oxidized low-density lipoprotein
MCP-1	Monocyte chemoattractant protein-1
IL-1β	Interleukin-1 beta
TNF-α	Tumor necro-sis factor-alpha
DHE	Dihydroethidium
BBDC	1-borono-3,5-benzenedicarboxylic acid
Ag NPs	Silver nanoparticles
POD	Peroxidase
GSSG	Oxidized glutathione
